# Draft Genome Sequence of the *Lactobacillus mucosae* Strain Marseille

**DOI:** 10.1128/genomeA.00841-15

**Published:** 2015-07-30

**Authors:** Fatima Drissi, Vicky Merhej, Caroline Blanc-Tailleur, Didier Raoult

**Affiliations:** Aix Marseille Université, URMITE, UM63, CNRS 7278, IRD 198, Inserm 1095, Marseille, France

## Abstract

*Lactobacillus mucosae* strain Marseille, isolated from stool samples of a child suffering from a malnutrition disorder called Kwashiorkor, produces bacteriocin and seems to have specific carbohydrate and lipid metabolisms different from those of other *Lactobacillus* organisms. The draft genome sequence of this strain is presented here.

## GENOME ANNOUNCEMENT

*Lactobacillus mucosae* is a heterofermentative lactic acid bacterium found in the gastrointestinal tract (1). It has been shown to have the ability to adhere to gastrointestinal mucus and to produce antimicrobial agents (2, 3), enabling the bacterium to colonize the gut efficiently and to inhibit the growth of pathogenic bacteria.

The genome of *L. mucosae* Marseille was sequenced using the MiSeq technology (Illumina Inc., San Diego, CA, USA) with the mate pair strategy. Illumina reads were assembled with SPAdes software (4, 5). Contigs obtained were combined by use of SSPACE (6) and Opera software v1.2 (7) helped by GapFiller v1.10 (8) to reduce the set. Noncoding genes and miscellaneous features were predicted using RNAmmer (9), ARAGORN (10), Rfam (11), Pfam (12), and Infernal (13). Coding DNA sequences (CDSs) were predicted using Prodigal (14), and functional annotation was achieved using BLAST+ (15) and HMMER3 (16) against the UniProtKB database (17). The BUR database and the KEGG automatic annotation server (KAAS) were used to annotate genes encoding bacteriocins (18) and to determine the metabolic profile of the genome (19), respectively.

The draft genome of *L. mucosae* Marseille consists of 12 contigs of sizes ranging between 2,027 and 539,623 bp. The genome consists of a single chromosome of 2,369,669 bp with 46.18% G+C content. Of the 2,282 predicted genes, 2,199 were protein-coding genes, 14 were rRNAs genes, and 69 were tRNA genes. A total of 1,535 genes (69.80%) were assigned as putative function, 62 genes (2.82%) were identified as ORFans (open reading frames [ORFs] with no detectable homology to other ORFs in the database), and the 481 remaining genes were annotated as hypothetical proteins (21.87%). A phylogenetic tree produced from the 16S rRNA genes revealed that strain Marseille is most closely related to *Lactobacillus mucosae* LM1 (NCBI reference sequence accession no. NZ_CP011013.1). A comparison between these two strains shows an identity of 97%, but a higher number of genes involved in carbohydrate transport and metabolism in strain Marseille than in LM1 (156 [8%] versus 131 [7%]).

The genome of strain Marseille includes genes encoding 12 different bacteriocins, ranging in size from 38 to 67 amino acids. Moreover, the *L. mucosae* Marseille genome has a specific mucus-binding protein (*mub*) gene, with significant homology (100% coverage and 100% similarity) to the *L. mucosae* LM1 *mub* gene. This gene is a common characteristic in *L. mucosae* species and has antimicrobial effects through cell surface protection (20). Interestingly, the Marseille genome encodes two enzymes that are not usually present in *Lactobacillus* genomes, an endoglucanase that participates in the degradation of cellulose to glucose and a maltooligosyl trehalose synthase that catalyzes the conversion of maltooligosaccharide into the nonreducing saccharide. Its genome also contains more genes involved in lipid transport and metabolism than other *Lactobacillus* genomes and has several genes encoding phospholipid phosphatase. Strain Marseille has a particular carbohydrate and lipid metabolism that may be attributable to the fact that it lives in the gastrointestinal tract of a malnourished host (M. Million, M. Tidjani Alou, S. Khelaifia, D. Bachar, J. C. Lagier, N. Dione, S. Brah, P. Hugon, V. Lombard, F. Armougom, J. Fromonot, C. Robert, C. Michelle, A. Diallo, A. Fabre, R. Guieu, C. Sokhna, B. Henrissat, P. Parola, and D. Raoult, submitted for publication).

### Nucleotide sequence accession number.

This whole-genome shotgun project has been deposited in the ENA under the accession no. CVQW00000000.
